# A Retrospective Study on the Efficacy of Subcutaneous Immunoglobulin as Compared to Intravenous Formulation in Patients with Chronic Lymphocytic Leukemia and Secondary Antibody Deficiency

**DOI:** 10.3390/curroncol30010022

**Published:** 2022-12-25

**Authors:** Andrea Visentin, Maria Chiara Molinari, Stefano Pravato, Alessandro Cellini, Francesco Angotzi, Chiara Adele Cavaretta, Valeria Ruocco, Silvia Imbergamo, Francesco Piazza, Giulia Proietti, Francesca Romana Mauro, Livio Trentin

**Affiliations:** 1 Hematology and Clinical Immunology Unit, Department of Medicine, University of Padova, 35128 Padova, Italy; 2 Hematology Unit, Department of Medicine of Systems (DIDAS), Azienda Ospedale Università Padova, 35128 Padova, Italy; 3 Hematology Unit, Department of Translational and Precision Medicine, Sapienza University, 00185 Rome, Italy

**Keywords:** secondary immunodeficiency, intravenous immunoglobulin, subcutaneous immunoglobulin, chronic lymphocytic leukemia, replacement therapy

## Abstract

Secondary antibody deficiency (SAD) is a common complication in chronic lymphocytic leukemia (CLL) which favors the development of life-threatening infections. Subcutaneous immunoglobulins (IG) (SCIG) have been proven to be as effective as intravenous immunoglobulin (IVIG) in primary immunodeficiencies. Since only a few studies investigated SCIG in secondary antibody deficiency, the aim of this study was to assess the efficacy and safety of SCIG or IVIG in CLL patients with secondary antibody deficiency. One hundred and sixteen CLL patients were recruited, 63% were males, and the median age was 68 years; 44% had bronchiectasis and 76% never smoked. Forty-nine patients received IVIG and 88 SCIG, including 28 patients who shifted from IVIG to SCIG. Despite similar baseline IgG levels, patients receiving SCIG achieved higher IgG after at least +6 months (*p* = 0.0009). We observed that SCIG can decrease the cumulative incidence of first (HR 0.39 *p* < 0.0001) and second (HR 0.56 *p* = 0.0411) infection more than IVIG. The effect was remarkable in that patients were able to reach at least 6 g/L of IgG after 6 months of treatments (*p* < 0.0001). Replacement therapies were well tolerated with less adverse events and a lower discontinuation rate in patients was managed with SCIG than IVIG. In this study we describe the clinical features of a large cohort of CLL with secondary antibody deficiency receiving IG. We demonstrated that SCIG are active and well tolerated drugs that allows to reach higher IgG levels and decrease the rate of infections better than IVIG, in particular when IgG levels reach 6 g/L.

## 1. Introduction

Chronic lymphocytic leukemia is the most common leukemia (CLL) in the adult Western population, and is characterized by the proliferation of clonal B lymphocytes expressing CD5, CD23 and CD200 [1,2]. The majority of patients are asymptomatic at diagnosis and do not require any treatment. Several studies have analyzed the genetic and molecular features of the disease, identifying subgroups of patients with shorter time to treatment, higher chemo-refractoriness and, finally, a lower overall survival. Thanks to these studies, key proteins involved in the survival of leukemic clones have been identified and new effective drugs have been developed, such as ibrutinib, venetoclax and obinutuzumab [3,4,5].

However, the most common causes of death for patients with CLL are infections and secondary cancers [2,3,6,7]. Secondary immunodeficiency is a common complication of patients with CLL due to abnormalities and impairment of both innate and adaptive immunity, which are intrinsic to the disease and further worsened by chemo-immunotherapies [1,8,9] and targeted therapies [6,10,11]. Secondary antibody deficiency accounts for only a small part of secondary immunodeficiency, including deregulation and deficiencies of T lymphocytes and monocytes [8]. On the other hand, secondary antibody deficiency is easy to identify, by measuring immunoglobulin levels in the peripheral blood, and can be rescued by immunoglobulin replacement therapy (IGRT) [1]. Secondary antibody deficiency might be present at CLL diagnosis or acquired during the follow-up even by treatment-naive patients.

IGRT is a valuable and effective option to increase IgG levels, to limit the use of antibiotics and to decrease the rate of severe and/or life-threatening infections in patients with primary and secondary immunodeficiency [12,13,14,15]. Subcutaneous immunoglobulins (IG) (SCIG) have been proved to be as effective as intravenous immunoglobulin (IVIG) in primary immunodeficiencies, but only a few studies investigated SCIG efficacy in secondary immunodeficiency [12,13,15].

In this study we described one of the largest populations of patients with CLL and secondary antibody deficiency who were treated with IGRT. Data on the efficacy, discontinuation and safety of SCIG or IVIG were reported and compared.

## 2. Methods

### 2.1. Study Design

This observational, retrospective, multicenter study aimed to evaluate the efficacy and safety of SCIG as compared to IVIG in patients with CLL and secondary antibody deficiency, according to clinical practice.

SCIG or IVIG were administrated to CLL patients with hypogammaglobulinemia and recurrent infections according to the Italian drug agency (AIFA) indications. The choice of IVIG or SCIG was made by the treating physician according to the patient’s status and institutions policies. IVIG were administrated every 3 or 4 weeks, depending on institution policy and availability. Serum IG levels (IgG, IgA and IgM) were recorded within 3 months before starting IG therapy (baseline), and after 3, 6 and 12 months of treatment.

Inclusion criteria were: age ≥ 18 years; diagnosis of chronic lymphocytic leukemia according to IWCLL 2016 criteria (either untreated, previously treated or on active treatment); has received IGRT till December 2019, either intravenous or subcutaneous, according to hospital policy; being able to sign the informed consent. Exclusion criteria were: any uncontrolled clinical condition, organ failure and/or laboratory abnormality; psychiatric diseases that impair the ability to give informed consent; concomitant immunosuppressive therapy (such as cyclosporine A, azathioprine, mycophenolate etc., but excluding CLL therapy); treatment with IGRT for less than 3 months.

### 2.2. Objectives of the Study

The primary objective was to compare the rate of bacterial or mycotic (proven or probable) infections of any grade before and after treatment with SCIG or IVIG. Secondary objectives were to compare between SCIG and IVIG the levels of serum IgG, IgA e IgM immunoglobulins after 3–6–12 months of therapy and/or at the last available follow-up; cumulative incidence of infections; the annual incidence of all grade infections and those graded as ≥3; rate and type of adverse events during IGRT; rates and causes of therapy discontinuation; the switch rate of IGRT type (i.e., from IVIG to SCIG and from SCIG to IVIG); bruising and bleeding in patients co-treated with ibrutinib (drugs approved for the treatment of chronic lymphocytic leukemia and associated with minor bleeding) [2,4]. Among patients who received SCIG we will perform the above-mentioned analysis comparing different subcutaneous drugs (i.e.g., 16% vs. 20% immunoglobulins concentrations).

### 2.3. Biological Markers

FISH analysis [16,17], TP53 mutation [18] and IGHV mutational status [19,20] were performed in all recruited patients in local accredited laboratories and their protocols are summarized in the Supplementary Materials. An IGHV gene sequence homology ≥98% was considered as unmutated (U-IGHV), opposed to mutated (M-IGHV) [21].

### 2.4. Statistical Analysis

Continuous variables were compared with the Mann–Whitney or Kruskal–Wallis test. Categorical variables were compared by the Chi-square test or the Fisher’s exact test, when appropriate. The cumulative incidence of first infection was calculated from the start of IGRT to the first infective event or last known follow-up (censored). The cumulative incidence second infection was calculated from the start of IGRT to the second infective event or last known follow-up (censored). Overall survival (OS) was calculated starting from the start of IGRT to death for any cause or last known follow-up. Survival analyses were performed by the Kaplan–Meier method and the Log-rank test was used to compare survival curves between groups. Hazard ratios (HR) with 95% confidential interval (CI) were also reported.

## 3. Results

### 3.1. Patients’ Features

The clinical and biological characteristics of patients are summarized in Table 1. Data of one hundred and sixteen CLL patients were analyzed; 63 (54%) were males, the median age at IGRT was 69 ± 10 years, 76% never smoked and 44% had bronchiectasis. The median years from CLL diagnosis and IGRT was 10 ± 7.5 years. Ninety-one per cent received at least one CLL-directed therapy (range 0–9) during the follow-up, including 90 patients (78%) who received anti-CD20 monoclonal antibodies, 29 patients (25%) who were treated with ibrutinib and 4 (3.4%) who were treated with venetoclax during their clinical course. From a biological point of view, 48% of patients harbored unmutated conformation of IGHV gene while 16% presented TP53 deletion or mutation. All the clinical and biological variables were balanced between IVIG and SCIG groups (Table 1); the median age at IGRT was higher for the SCIG group consistent with the fact that some patients shift from IVIG to SCIG (see afterward). After a median follow-up of 44.6 months from the beginning of IGRT, 22 (18.9%) patients died due to infections, 9 (7.8%) of Richter syndrome transformation [22], 5 (4.3%) of CLL progression and 9 (7.8%) other diseases such as second primary malignancies or cerebral hemorrhage. The estimated median OS was 94.7 months (Supplementary Figure S1A).

### 3.2. Immunoglobulin Levels

**Figure 1:**
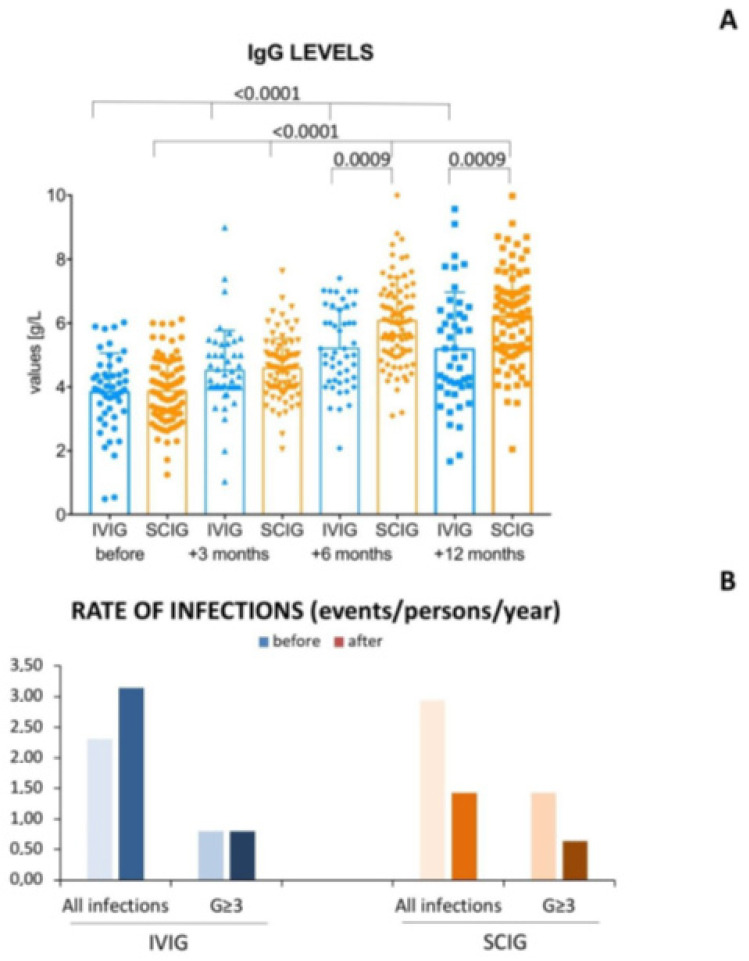
Histograms of IgG levels and infection rates. In the upper panel (**A**) there is a histogram reporting the serum IgG levels at baseline, after 3, 6 and 12 months of intravenous immunoglobulins (IVIG) or subcutaneous immunoglobulin (SCIG). Kruskal–Wallis test was used to analyse IgG levels in patients receiving IVIG and SCIG at different time points. Mann–Whitney test was used to compare IgG at same time point between patients receiving IVIG and SCIG. SCIG allowed patients to reach higher IgG trough levels than IVIG after at least 6 months of treatment. In particular, at month +6, IgG > 6 g/L was achieved by 33.2% and 52.3% of patients with IVIG and SCIG (*p* = 0.0322), respectively. In the lower panel (**B**) are shown the rates, expressed as events/person/year, of all infections and grade ≥ 3 (G3) events before and after IVIG and SCIG.

Forty-nine patients received IVIG and 88 SCIG (41 subjects the 20% IG formulation and 47 the 16% drug), including 28 patients who shifted from IVIG and SCIG. The median monthly IG dose administrated was 15.0 ± 3.9 g/L and 18.8 ± 2.9 g/L for IVIG and SCIG, respectively (*p* < 0.0001). Overall IgG levels increased with both IVIG and SCIG (*p* < 0.0001, Figure 1A). However, despite similar baseline (3.8 ± 1.2 g/L vs. 3.9 ± 1.0 g/L, *p* = 0,7534) and +3 months (4.5 ± 1.3 g/L vs. 4.6 ± 0.9 g/L, *p* = 0.5028) IgG levels, patients receiving SCIG were able to achieve higher IgG levels at +6 (6.0 ± 1.4 g/L vs. 5.2 ± 1.2 g/L, *p* = 0.0009) and +12 months (6.2 ± 1.5 g/L vs. 5.2 ± 1.8 g/L, *p* = 0.0009) than IVIG (Figure 1A). Graphics of IgG mean, min, max and interquartile values are provided in Supplementary Figure S1B. IgA and IgM levels remained stable during the follow-up (Supplementary Figure S1C,D). The type of SCIG, namely Hizentra^®^ (20% formulation), Octanorm^®^ (16% formulation) or Subcuvia^®^ (16% formulation), did not influence the IgG levels at the timepoints (Figure S1E). After 6 months of replacement therapy, IgG > 6 g/L was achieved by 33.2% and 52.3% of patients with IVIG and SCIG (*p* = 0.0322), respectively.

### 3.3. Incidence of Infections

Overall, 254 infections occurred, of which 94% were bacterial and 6% mycotic. The most common infections involved the upper respiratory tract (61%), pneumonia cases (35%) and sepsis (26%). Remarkably, the incidence of infections increased from 2.31 to 3.14 events/patient/year before and during IVIG treatment, while the incidence of infective events decreased from 2.59 to 1.43 events/patient/year before and during the SCIG therapy (Figure 1B). Considering the incidence of grade ≥3 infections, this remained stable with IVIG (0.80 events/patient the year before and during IVIG), while it decreased from 1.43 to 0.64 with SCIG (Figure 1B).

### 3.4. Cumulative Incidences of First and Second Infection

**Figure 2:**
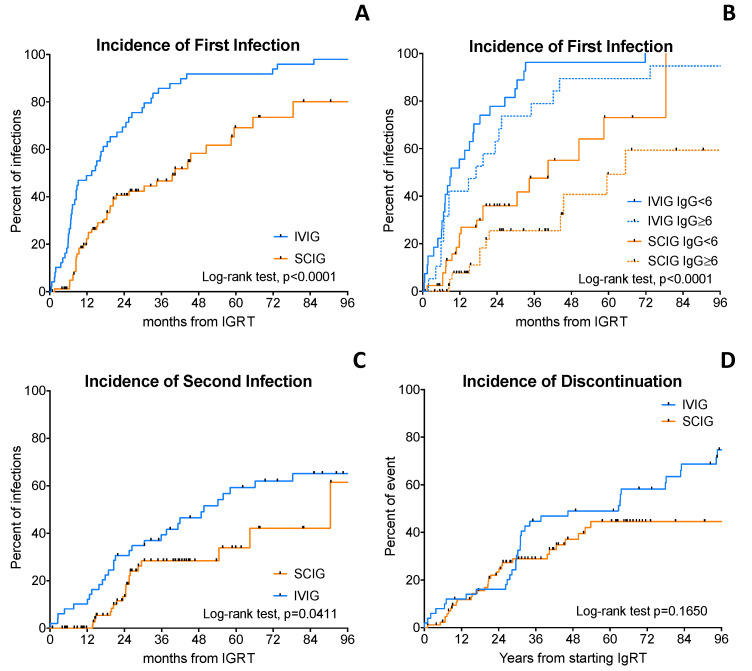
Cumulative incidence of infections and discontinuation. The upper panel on the left (**A**) shows the Kaplan–Meier curves of time to first infection in patients treated with intravenous immunoglobulins (IVIG) or subcutaneous immunoglobulin (SCIG). The upper panel on the right (**B**) shows the Kaplan–Meier curves of time to first infection in patients IVIG or SCIG, according to serum IgG levels. Patients achieving at least 6 g/L of IgG display a longer time to the first infection. In the lower-left panel (**C**) is shown the Kaplan–Meier curves of time to second infection in patients treated with IVIG or SCIG. In the lower-right panel (**D**) is shown the Kaplan–Meier of the cumulative incidence of discontinuation in patients treated with IVIG or SCIG. Log-rank test was used to compared survival curves.

Subsequently, we analyzed the cumulative incidences of first and second infection in patients receiving IGRT. We observed that the cumulative incidence of first infections was occurred later for patients receiving SCIG than IVIG (Figure 2A). After a median duration of IGRT of 4.1 years (3.7 years for IVIG and 4.8 year for SCIG), the 4-year cumulative incidence of first infection was 91% and 58% with IVIG and SCIG, respectively (*p* < 0.0001, Figure 2A). SCIGs were able to decrease the risk of first infection by almost 60% (hazard ratio 0.39, 95% CI 0.25–0.61). In particular, we found that median cumulative incidence of first infection was later for patients who achieved +6 months IgG > 6 g/L than for those who did not; it was 9.2 months vs. 17.3 months with IVIG and 40.5 months vs. 65.5 months with SCIG (*p* < 0.0001, Figure 2B). Interestingly, also the cumulative incidence of second infections was improved with SCIG. The 4-year cumulative incidence of second infection was 46.5% and 28.4% with IVIG and SCIG, respectively (HR 0.56, 95% CI 0.32–0.00, *p* = 0.0411, Figure 2C).

### 3.5. Discontinuation Rate

Seventy-one % of subjects discontinued IVIG therapy as compared to 36% with SCIG (*p* = 0.0002). The estimated 3-year cumulative incidence of IGRT discontinuation was 44% vs. 29% for IVIG and SCIG, respectively (Figure 2D, *p* = 0.1650). The main reason of discontinuation of IVIG was the shift to SCIG (83%) mainly due the presence of infective events during IVIG therapy, followed by death of the patients (13%) and infusion-related reactions (4%). Conversely, the reasons of SCIG discontinuation were death (81% of the patients who discontinued and 29% of patients treated with SCIG) and intolerance due to recurrent local reactions (in 19% of the patients who discontinued and 6.8% of patients treated with SCIG). Twenty-eight patients shifted from IVIG to SCIG, while none did the opposite. The main reason of shifting from IVIG to SCIG was mainly due to recurrences of infections and infusion-related reactions. Remarkably, patients who shifted from IVIG to SCIG were able to achieve higher IgG levels after at least 6 months of treatment than patients who continued to receive IVIG (Supplementary Figure S1F).

### 3.6. Safety

IGRTs were well tolerated but infusion-related adverse events were more common with IVIG than SCIG (20.8% vs. 5.6%, *p* = 0.0193). The most common adverse events in patients receiving IVIG were infusion-related reactions (16.3%) characterized by fever in 16.3% of patients, chills (12.2%), rash (7.2%), headache/dizziness/nausea (8.2%) and dyspnea (6.1%). Fifty-one per cent of patients required premedication with steroids and antihistamines before the infusion of IVIG.

During SCIG, adverse events were usually local mild rash/bruising (3.4%), and only two patients (2.3%) developed fever. Overall, the incidence and intensity of infusion-site reactions decreased over time. It is noteworth that we did not observe any case of infection at the site of subcutaneous infusion. Bruising did not increase during concomitant SCIG and ibrutinib (which is known to be associated with a mild hemorrhagic risk).

## 4. Discussion

Secondary antibody deficiency is a common, but often overlooked, complication in patients with CLL [15,23,24]. In this study, we reported on one of the largest populations of CLL patients who received IGRT. Comparing the subcutaneous formulation with IVIG, patients receiving the SCIG achieved (i) higher IgG trough levels, (ii) fewer infections, (iii) longer time to the first and second infection and (iv) fewer adverse events.

IVIG have been extensively investigated in patients with CLL [1,2,24,25]. Despite infections being the main cause of death in CLL patients, and older studies showing that IVIG were able to decrease the rate of infective events, IVIG did not provide any survival improvement and few data existed on SCIG [25,26,27]. Treatments of CLL patients have been extensively improved since those studies. The new anti-CD20 monoclonal antibodies and targeted therapies towards BTK or BCL2, either alone or in combination, proved to be more active, better tolerated than chemoimmunotherapy, and to increase the survival of CLL patients [1,2,3]. Despite some of these targeted therapies being able to cause hypogammaglobulinemia [1,3], only a few studies investigated the IGRT in patients treated in the current era. In our study we document that IGRT is a valid strategy to decrease the rate of infection. Furthermore, we observed that SCIG were better than IVIG in decreasing the rate of infection and to delay both the first and the second infective event. We explain this aspect by the fact that patients treated with SCIG received a higher monthly dose of IGRT, more patients achieved at least 6 g/L of IgG after 6 months and thus less patients developed infection.

Interestingly, Cinetto et al. [12], investigated IGRT strategies in a large cohort of patients with primary and secondary antibody deficiencies, including 55 (42%) CLL patients. The authors found that patients with secondary antibody deficiency required lower SCIG dosage and lower IgG trough level to decrease infective events at the steady state. SCIG tolerance was comparable in patients in patients with secondary and primary antibody deficiencies. In a French observational study describing the use of octagam^®^ (IVIG drug) or gammanorm^®^ (SCIG drug) in secondary immunodeficiencies associated with hematological malignancies, 54 (33.8%) patients had CLL [13]. Compared to baseline, IGRT increased serum IgG by 3.4 g/L and decreased the frequency and severity of infections. IGRT was discontinued in 9% of patients [13]. Similarly, in our study which recruited a higher number of CLL patients and used different drugs, SCIG allowed achievement of higher IgG levels, to decrease and delay the risk of infections with an acceptable safety profile.

Recently Innocenti et al. [14] reported on 10 CLL patients who received a flat dose of 10 g/month of SCIG independently from body weight. All patients tolerated the therapy well and experienced an increase of IgG levels, which remained stable through time. The authors also suggested that SCIG are particularly advantageous in the COVID-19 era, because the self-administration at home allowed a decrease in hospital admissions and treatment expenditures [14]. CLL patients are at higher risk of developing severe and life-threating SARS-CoV2 infections [28,29], and display lower serum conversion rates after vaccinations [30] (including SARS-CoV2 vaccine [31]); thus, all strategies to limit the spread of infections should to be considered [32,33].

Switching between IVIG and SCIG has been reported in patients with primary immunodeficiency. In the study of Canessa et al. [34] 19 patients switched, with a decrease of number of infusions but no difference in terms of effectiveness, safety and satisfaction. In our study on secondary antibody deficiency, we showed that patients who shifted from IVIG to SCIG were able to achieve higher IgG levels.

Recombinant human hyaluronidase-facilitated SCIG (fSCIG) have been approved for CLL patients with secondary antibody deficiency. Advantages of fSCIG include fewer needle punctures, longer infusion intervals and improved adverse effects profile compared to IVIG [35]. Limited real-life experiences exist concerning the fSCIG in CLL patients and its use deserves further investigation [36,37].

The main limitation of the study is its retrospective nature. Patients were treated according to clinical practice guidelines and institutional policies. In order to decrease the selection bias, we asked the investigators to report all the CLL patients treated with SCIG or IVIG. We analyzed the reported data and performed computerized manual consistency checks on each case report form.

## 5. Conclusions

We herein analyzed the clinical features of a large cohort of CLL with secondary antibody deficiency receiving IGRT. We demonstrated that SCIG are active and well tolerated drugs that allow patients to reach higher IgG levels, while decreasing the rate of infection better than IVIG, particularly when IgG levels reach 6 g/L.

## Figures and Tables

**Table 1:** Clinical and biological features of patients.

Variables	All Patients	IVIG	SCIG	*p* Values
Number of cases	116	49	88	-
Median age (years)	69 ± 10.1	67 ± 8.2	70 ± 8.0	0.0460
Gender				
Male/Female	63 (54%)/53 (46%)	25 (51%)/24 (49%)	49 (56%)/39 (44%)	0.7208
Binet stage A/B/C	77 (66%)/27 (23%)/12 (10%)	28 (57%)/14 (29%)/7 (14%)	60 (68%)/20 (23%)/8 (9%)	0.4053
Years from CLL diagnosis to IGRT	10.3 ± 7.5	9.1 ± 7.3	11.04 ± 7.53	0.0853
Baseline IgG (g/L)	3.9 ± 1.1	3.8 ± 1.2	3.9 ± 1.0	0.7534
Months from IgG test and start IGRT	1.1 ± 1.1	1.2 ± 1.1	0.9 ± 1.0	0.7610
Never smoked	88 (76%)	39 (80%)	70 (80%)	0.9998
Bronchiectasis	51 (44%)	19 (39%)	37 (42%)	0.7214
IGHV status *				0.8405
Mut./Unmut.	47 (52%)/44 (48%)	19 (53%)/17 (47%	39 (55%)/32 (45%)	
FISH *				0.9702
13q-/normal	44 (44%)/19 (19%)	18 (44%)/6 (15%)	34 (43%)/14 (18%)	
+12/	9 (9%)/	4 (10%)	9 (11%)/	
11q-/17p-	16 (16%)/12 (12%)	6 (15%)/5 (13%)	15 (19%)/8 (10%)	
TP53 Abnormalities *	16 (16%)	7 (15%)	12 (15%)	0.7918
Previous therapies for CLL	105 (91%)	44 (90%)	77 (88%)	0.7868
Previous therapies, median (IQR)	3 (2–6)	3 (2–6)	3 (2–7)	0.7091
anti-CD20 mAb	90 (78%)	36 (73%)	70 (80%)	0.5232
Ibrutinib therapy	29 (25%)	19 (39%)	23 (26%)	0.1755
Venetoclax therapy	4 (3.4%)	2 (4%)	4 (5%)	0.9999
Died	42 (36%)	15 (31%)	29 (33%)	0.9999

* IGHV status available for 91 patients, FISH analysis available for 100 patients and TP53 abnormalities available for 100 patients. Anti-CD20 mAb = anti-CD20 monoclonal antibodies (such as rituximab, ofatumumab or obinutuzumab). IQR = interquartile range.

## Data Availability

The datasets generated and analyzed during the current study are not publicly available due to data protection and lack of consent from patients. Access to data is strictly limited to the researchers who have obtained permission for data processing.

## Supplementary Materials

The following supporting information can be downloaded at: https://www.mdpi.com/article/10.3390/curroncol30010022/s1, Figure S1: Overall survival and immunoglobulin histograms.
