# Supplementary material for: A Retrospective Study on the Efficacy of Subcutaneous Immunoglobulin as Compared to Intravenous Formulation in Patients with Chronic Lymphocytic Leukemia and Secondary Antibody Deficiency
Source: Curr Oncol. 2022 Dec 25;30(1):274–83. doi: 10.3390/curroncol30010022 (PMC9857433; doi:10.3390/curroncol30010022)
Supplement: Supplementary file 1 [file curroncol-30-00022-s001.zip › curroncol-2019353-supplementary.pdf]

SUPPLEMENTARY METHODS

IGHV mutational status

Analysis of the IGHV mutational status was performed within 12 months from diagnosis on peripheral blood CLL cells from fresh samples or frozen purified CLL cells harvested in DMSO. RNA was extracted from 2x106 B cells using the RNeasy™ Total RNA kit (Qiagen, Hilgen, Germany) and reverse transcribed using the SuperScript™ Preamplification System for first-strand cDNA synthesis (Life Technologies, Carlsbad, CA). The CLL B-cell HV gene family was assigned as previously described(17,18). HV gene sequences were determined by amplifying 5µl of the original cDNA using the appropriate HV leader and HC primers. PCR products were directly sequenced after purification with the Wizard PCR Preps (Promega, Madison, WI) using an automated genetic analyzer (3130 ABI Applied Biosystems, Foster City, CA, USA). Sequences were analyzed using the IMGT/VQUEST and BLAST softwares(17,18) to detect VDJ junction. Cases with a sequence homology <98 from the corresponding germline gene were considered as mutated (M-IGHV), and those with a homology ≥98% as unmutated (U-IGHV)(17,18).

Cytogenetics by fluorescence in situ hybridization (FISH) and TP53 mutations

FISH was performed on standard cytogenetic preparations from peripheral blood(14). The slides were hybridized with the multicolor probe set LSI p53/LSI ATM and LSI D13S319/LSI 13q34/ CEP12 and RP11-177O8 according to the manufacturer's instructions(15). Three hundred interphase nuclei were analyzed for each probe and the cut-off for positive value was 10% for deletion of 11q22.3 (ATM), 17p13.1 (TP53) loci and 13q14.3 (D13S319), and 5% for trisomy 12. TP53 gene sequencing was performed according to ERIC guideline assessing exons 4-10; if negative exons 2, 3 and 11 were also investigated(16).

LEGEND TO SUPPLEMENTARY FIGURES

Figure S1. Overall survival and immunoglobulin histograms. In the upper left panel (A) there is a Kaplan-Meier curve of overall survival of all CLL patients after immunoglobulin replacement therapy (IGRT). In the upper right panel panel (B) the a box and whiskers plot reporting the serum IgG levels at baseline, after 3, 6 and 12 months of intravenous immunoglobulins (IVIG) or subcutaneous immunoglobulin (SCIG). In the middle panel, there is on the left (C) a histogram of IgA and IgM levels in patients receiving intravenous immunoglobulin (IVIG), and on the right (D) a histogram of IgA and IgM levels in patients receiving subcutaneous immunoglobulin (SCIG). In the lower-left (E) panel there is a histogram of IgG levels in patients receiving different SCIG drugs (Hizentra®, Octanorm® or Subcuvia®). In the lower-right panel (F) there is a histogram of IgG levels in patients who shifted from IVIG to SCIG. Kruskal Wallis test used to analyze IgG levels in patients receiving different SCIG products. Mann-Whiney test was used to compared immunoglobulin levels at same time point between patients receiving IVIG and SCIG.

Figure S1

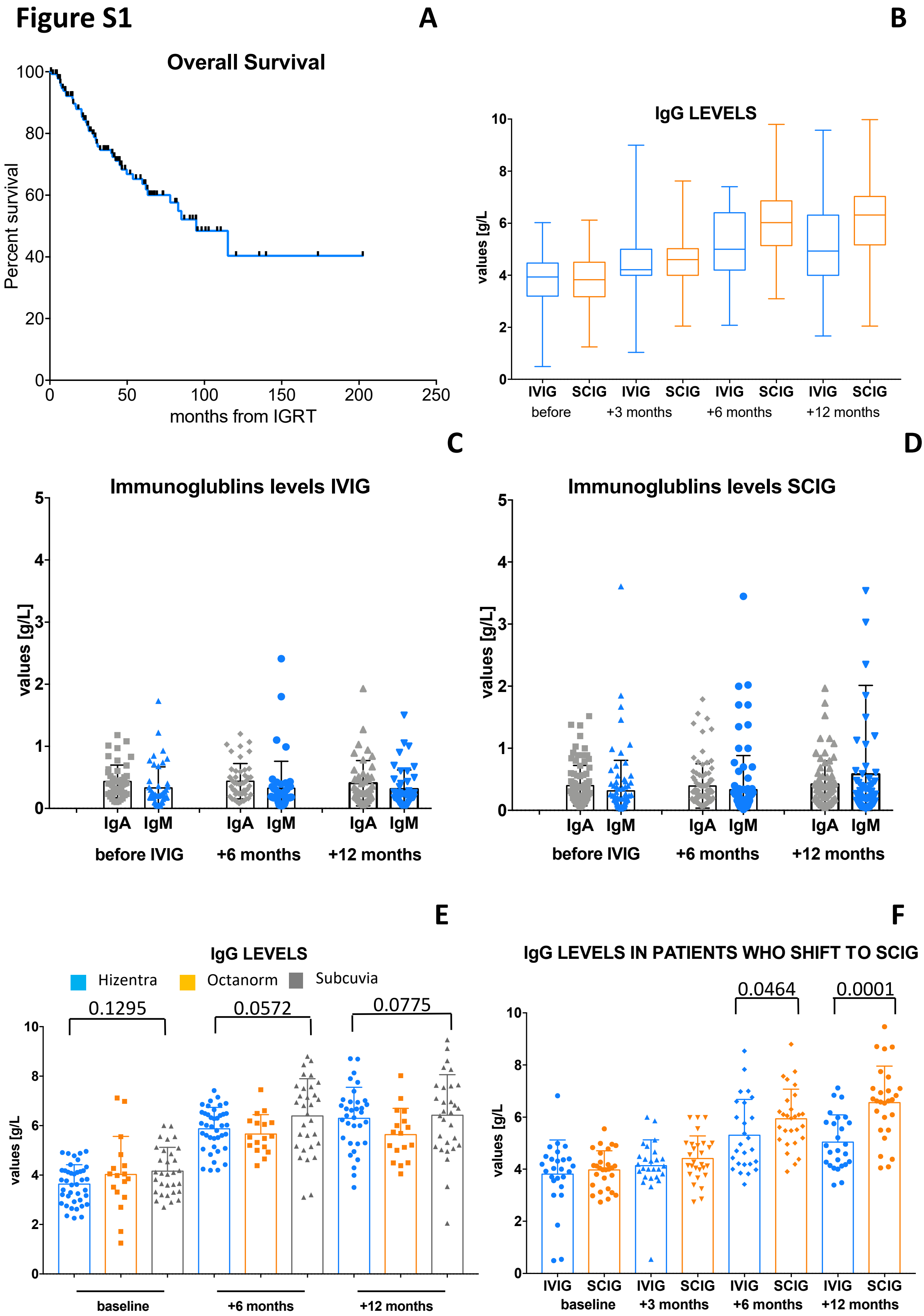
A
Overall Survival
Percent survival
months from IGRT
B
IgG LEVELS
values [g/L
IVIG SCIG IVIG SCIG IVIG SCIG IVIG SCIG
before +3 months +6 months +12 months
C
Immunoglublins levels IVIG
values [g/L]
IgA IgM IgA IgM IgA IgM
before IVIG +6 months +12 months
D
Immunoglublins levels SCIG
values [g/L]
IgA IgM IgA IgM IgA IgM
before IVIG +6 months +12 months
E
IgG LEVELS
Hizentra Octanorm Subcuvia
0.1295 0.0572 0.0775
values [g/L
baseline +6 months +12 months
F
IgG LEVELS IN PATIENTS WHO SHIFT TO SCIG
0.0464 0.0001
values [g/L
IVIG SCIG IVIG SCIG IVIG SCIG IVIG SCIG
baseline +3 months +6 months +12 months
